# Coping strategies and perceived burden among caregivers of frail elderly with mental disorders attending teaching hospital: A cross-sectional study

**DOI:** 10.1371/journal.pone.0349689

**Published:** 2026-05-27

**Authors:** Anjana Maharjan, Shobha Laxmi Bajracharya

**Affiliations:** 1 MN (Psychiatric Nursing), Lalitpur Nursing Campus, School of Nursing and Midwifery, Patan Academy of Health Sciences, Lalitpur, Nepal; 2 Lalitpur Nursing Campus, School of Nursing and Midwifery, Patan Academy of Health Sciences, Lalitpur, Nepal; The Chinese University of Hong Kong, HONG KONG

## Abstract

Caregivers of frail elderly with mental illness undergo various practical problems, however, adaptive coping skills help to reduce perceived caregiver burden. This study aimed to identify the coping strategies and perceived burden among caregivers of frail elderly with mental disorders. A cross-sectional study was conducted among 129 conveniently selected caregivers of frail elderly with mental disorders at Patan Hospital. Data were collected using a face-to-face interview technique. The Nepali versions of Brief Cope (28 items) and Zarit Burden Interview (ZBI-22 items) were used to assess coping skills and burden respectively. Descriptive and statistical tests (Chi-square, Pearson’s correlation, and Logistic regression) were used to inferential analysis using SPSS version 16. Caregivers most commonly used problem-focused (29.19 ± 3.09; p = 0.112) and emotion-focused (33.16 ± 4.91; p = 0.057) coping strategies. Nearly half of the caregivers (48.1%) reported little/no burden, while 15.5% reported severe burden in terms of personal strain (35.96 ± 25.96) and role strain (30.13 ± 33.54). Gender (𝑋^2^ = 6.630; *p =* 0.01), type of family (𝑋^2^ = 8.420; *p =* 0.004) and employment status of caregivers (𝑋^2^ = 4.069; *p* = 0.044) were significantly associated with the level of perceived burden. The study found no significant correlation between coping strategies and burden, however, the direction of correlation suggested an inverse correlation between problem-focused coping and burden (r = −0.141; *p* = 0.112). Effective coping strategies, especially problem-focused coping strategies, can help minimize caregiver burden. However, caregivers living in nuclear families are more likely to experience higher levels of burden. These findings highlights the need for targeted support and counseling services to assist caregivers and reduce their overall burden.

## Introduction

Caregiver burden is the term that refers to the significant stress and challenges experienced by caregivers or family members of individuals with mental disorders [1]. The disease burden of mental disorders is documented to be high among the population of frail elderly individuals [2]. A systematic review of multiple studies conducted across Nepal highlighted a significant prevalence of depressive symptoms among frail elderly. These symptoms were notably widespread across diverse environments, including community, aged-care facilities, and hospital settings [3].

A member with a severe mental disorder can have a significant impact on the entire family system [4,5]. Family caregivers of people with mental disorders are the major support system in Nepal and most of the North West [6]. For instance, caregivers use adaptive (positive) or maladaptive (negative) coping mechanisms to deal with issues brought on by family members’ mental disorder [7–9]. Coping refers to process by which a caregiver solves problems, makes decisions, and relieves tension [10,11]. Coping strategies can also be analyzed as problem-focused coping, emotion-focused coping and avoidant coping [10].

Effective and adaptive coping mechanisms have been shown to reduce perceived caregiver burden [12,13]. A cross-sectional study involving family caregivers of patients with chronic schizophrenia and bipolar disorder type I revealed a significant relationship between the challenges of care giving and the methods used to manage them. Specifically, higher levels of caregiver burden were associated with a lower utilization of problem-focused coping strategies across both patient groups [13]. Another study conducted among caregivers of Chinese patients with dementia demonstrated that problem-focused and emotion - focused coping strategies are beneficial in terms of reducing caregiver burden [14].

Likewise, failure to use effective coping strategies maximize burden. It has been reported that the majority of caregivers experience a moderate burden in the course of their duties [15–17]. A descriptive cross-sectional study among caregivers of patients, including frail elderly with mental disorders in Chitwan Medical College, Nepal, showed that caregivers perceived moderate to severe burden (47.1%), mild to moderate (41.2%) and minimal burden (11.8%) [18].

A cross-sectional study conducted among 1163 caregivers of urban frail elderly, including those with neuropsychiatric or cognitive disorders in India, highlighted that the caregiver experienced behavioral, emotional, cognitive problems as well as difficulties in self-care and functioning. Additionally, caregivers also experienced strain in terms of dangers, embarrassment, sleep, coping, depression, worry, household routine, frustration, enjoyment of role, holidays, finance, health, and attention due to their care-giving responsibilities [19]. Families who are already struggling with a variety of daily issues that are distressing all aspects of their lives, they may experience personal and role strain while providing care. Personal strain means’ how personally stressful the experience is’; and role strain is’ the stress due to role conflict or overload [20–22]. Thus, studying coping strategies could be a useful way of generating information that can help guiding stress management strategies. This research aims to provide preliminary data on these issues and to assess coping strategies and perceived burden among caregivers of frail elderly with mental disorders.

## Materials and methods

### Study design, setting, period, and sample size

A descriptive cross-sectional study was conducted among caregivers of frail elderly with mental disorders at the Psychiatric Out-Patient Department (OPD) and In-Patient Department (IPD)of Patan Academy of Health Sciences (PAHS), Nepal. The overall study duration lasted from November 2022 through April 2024, with the primary data collection phase conducted between August 13 and September 29, 2023.Although data collection was initially scheduled for six weeks duration, the timeline was extended by an additional week to ensure the target sample size was reached. The calculated sample size for the study was 159. However, only 129 caregivers who met the inclusion criteria during the study period were included in the final analysis. A total of 159 caregivers were initially approached for participation. Among them, one caregiver refused to participate. Two caregivers initially provided incomplete responses to the questionnaire due to being called for their psychiatric OPD appointment; however, they were later re-approached and completed the questionnaire with their consent. Additionally, 29 caregivers were excluded because they did not meet the inclusion criteria. Of these, two caregivers did not meet the required age criterion (≥18 years), while the remaining 27 caregivers did not meet the inclusion criterion related to the duration of care-giving (minimum six months).

Thus, using a convenient sampling technique, 129 participants were included in the final analysis after applying inclusion criteria (caregivers aged ≥18 years providing care for at least six months to a frail elderly person aged ≥60 years).

### Data collection procedure

At first, the respondents (i.e., caregivers) were evaluated for inclusion criteria to be met.

Then the respondents were explained about the objectives of the study and time duration (25–30 minutes) taken for data collection. Informed written consent was taken from each respondent before the interview using generic PAHS format. Respondents’ right was respected and protected considering the rights to full information disclosure, justice, right to fair treatment, voluntary inclusion and withdraw from study anytime. Nobody was forced to participate in the study. Face to face interview was conducted using structured interview questionnaire in Nepali language in Psychiatric OPD (n = 126) and IPD (n = 3). Privacy of respondents was maintained by taking the interview in separate place of waiting area according to the comfort of respondents with one to one approach as per the space available in the Psychiatric OPD and IPD. Three to five respondents were interviewed daily each taking for about 25–30 minutes duration for data collection in psychiatric IPD and in OPD except for Wednesday and Saturday. Psycho-education was provided to caregivers in need. Caregivers were also recommended to attend counseling sessions which would help them to minimize the burden of care-giving. Confidentiality was maintained all the time during study period by coding the questionnaire, storing printed copies in a personal locked cabinet, saving file on password protected file by not sharing data to any other than research guide team.

### Study tool

Data were collected using a three part structured instrument:

Part-I: Socio-demographic and clinical questionnaire: A self developed structured questionnaire to assess socio-demographic and clinical variables. These included age, gender, marital status, type of family, education and employment status of caregiver, and clinical details of care recipient.

Part-II: The Brief COPE Inventory (Nepali version): A 28- item tool measured on a 4-point Likert scale (1–4) such as 1 = never does it, 2 = does it a few times, 3 = does it mostly but not always, and 4 = does it always. Each of the 14 scales is comprised of 2 items; total scores on each scale ranges from 2 (minimum) to 8 (maximum). The score ranges from 28–112 [23]. The Cronobach’s alpha for the original subscales of the Brief COPE ranged from 0.50 (venting) to 0.90 (substance use) [24]. The Nepali version of Brief COPE Inventory – 28 items was analyzed as Problem focused, Emotion focused and Avoidant coping [25,26].

Part-III: Zarit Burden Interview (ZBI – 22, Nepali version): A 22-item scale was used to measure level of perceived burden. The scale measured on a 5 – point Likert scale as 0 (never), 1 (rarely), 2 (sometimes), 3 (quite frequently) and 4 (nearly always). The instrument has a total score of 88 [27]. The Cronbach’s alpha value for ZBI items was reported as 0.93 and the intra-class correlation coefficient for the test retest reliability of ZBI was 0.89 [28]. The subscales of perceived burden includes personal strain (α = .80), and role conflict (α = .81) [22]. Cronbach’s alpha for personal strain and role strain was 0.89 and 0.77 respectively [20].

### Study variables

The primary dependent variable is the perceived burden among caregivers. Independent variables include socio-demographic and clinical variables (e.g., age, gender, marital status, type of family, education and employment status of caregiver, current mental disorder of frail elderly, duration of mental disorder, relationship with care recipient (frail elderly), and duration of care giving) and coping strategies (Problem –focused, Emotion- focused, and Avoidant coping).

### Ethical approval and informed consent

Ethical approval was obtained from the Institutional Review Committee (IRC) [Ref: PNM2307041759, PNM2307041759.A1] of Patan Academy of Health Sciences (PAHS). All participants provided written informed consent after being briefed on the study’s objectives, voluntary nature, and their right to withdraw at any time. Confidentiality was maintained through coded questionnaires and password protected data storage.

### Statistical analysis

The collected data were checked for completeness, organized, coded, and entered into the Statistical Package for the Social Science (SPSS) version 16 for analysis. Both descriptive and inferential statistics were used, considering 95% confidence interval, 5% permissible error and a significance level of p < 0.05.

Descriptive statistics (frequency, percentage, median and quartile deviation) were used for socio-demographic characteristics and clinical variables. Inferential analysis included Chi-square test to determine association between level of perceived burden and socio-demographic variables. Variables showing significant association (gender, employment status, and type of family) were further analyzed using logistic regression after meeting the necessary assumptions, in order to determine the effect of independent variables on the dependent variables (level of perceived burden).

For this analysis, the level of burden was categorized as no or little to mild burden and moderate to severe burden. Pearson’s product moment correlation was used to examine the relationship between coping strategies and burden after meeting the assumption of normality. No outliers were identified in the dataset. The analyzed data and results were presented using tables and figures.

## Results

Table 1 shows that majority of caregivers (55.0%) belonged to the 19–39 years age group. Regarding gender distribution, 54.3% were male and 45.7% were female. Most caregivers (75.2%) were married, and 90.7% were literate. More than half (66.0%) of respondents belonged to joint families, and 58.0% were unemployed. Among the caregivers, the majority (52.7%) had a parent-child relationship with the care recipient (frail elderly). Additionally, 50.4% of caregivers had been providing care to frail elderly for two years or less (≤ 2 years).

**Table 1:** Socio-demographic Characteristics of Respondents (Caregivers).

Variables	Frequency (n)	Percentage (%)
**Age(in completed years)**		
19-39	71	55.0
40-64	44	34.1
≥65	14	10.9
Median(Q1,Q3):38(30.00,51.50);Range:19–77		
**Gender**		
Male	70	54.3
Female	59	45.7
**Marital Status**		
Unmarried	30	23.3
Married	97	75.2
Widow/Widower	2	1.5
**Educational Status**		
Illiterate	12	9.3
Literate	117	90.7
**Type of Family**		
Nuclear	40	31.0
Joint	85	66.0
Extended	4	3.0
**Employment Status**		
Employed	54	42.0
Unemployed	75	58.0
**Relationship with care recipient**		
Spouse	24	18.6
Children	68	52.7
Others*	37	28.7
**Duration of care-giving to frail elderly**		
≤2years	65	50.4
>2years	64	49.6
Median(Q1,Q3):1.5(0.58–3.00); Range:0.5–28years		

N = 129.

Note: *includes daughter-in-law, son-in-law, siblings and grandchildren.

Table 2 shows that 72.9% care recipients belonged to the 60–75 years age group. The majority (64.3%) were female, and 81.4% were married. Nearly three-fourths (73.6%) of frail elderly had non-organic disorders. More than half (55.0%) had been experiencing mental disorder for more than two years.

**Table 2:** Demographic and Clinical Variables of Care recipients.

Variables	Frequency (n)	Percentage (%)
**Age(in completed years)**		
60-75years	94	72.9
≥75years	35	27.1
Median(Q1,Q3):66(60.00,75.00); Range:60–92		
**Gender**		
Male	46	35.7
Female	83	64.3
**Marital Status**		
Married	105	81.4
Divorced	2	1.6
Widow/Widower	22	17.0
**Current Mental Disorder** ^**α**^		
Organic disorder	34	26.4
Non-organic disorder	103	79.9
**Duration of Mental Disorder**		
≤2years	58	45.0
>2years	71	55.0
Median(Q1,Q3):1(1,2); Range:1–2		

N = 129.

Note: α-Multiple responses.

Note: Organic disorders included dementia, delirium, organic psychosis, organic mood disorder, and seizure disorder. Non-organic disorders included anxiety disorders, somatoform disorders, mood disorders (mania, depression, bipolar affective disorder and dysthymia), and alcohol use disorder (alcohol dependence syndrome with uncomplicated withdrawal and alcohol dependence with complicated withdrawal).

Table 3 shows that among the 129 respondents, 48.1% reported little or no burden, 24.0% reported mild to moderate burden, 12.4% reported moderate to severe burden and 15.5% reported severe burden.

**Table 3:** Level of Perceived Burden of Respondents.

Level of Burden (Score)	Frequency (n)	Percentage (%)
Little or no burden(0–20)	62	48.1
Mild to moderate burden(21–40)	31	24.0
Moderate to severe burden(41–60)	16	12.4
Severe burden(61–88)	20	15.5
Median(Q1,Q3):21(10,45)		

N = 129.

Table 4 shows the association between level of perceived burden and selected characteristics of respondents and care recipients. Bivariate analysis using the Chi- Square test revealed that level of perceived burden was significantly associated with caregiver’s gender(𝑋^2^ = 6.630; p = 0.01), type of family(𝑋^2^ = 8.420; p = 0.004), and employment status (𝑋^2^ = 4.069; p = 0.044). In the multivariable logistic regression model, only type of family remained a statistically significant independent predictor of burden. Caregivers residing in nuclear families had 2.8 times higher odds of experiencing moderate to severe burden compared to those living in joint families (AOR = 2.86, 95% CI: 1.24–6.61; p = 0.013). While gender and employment were significant in the bivariate analysis, their significance was not maintained in the adjusted model (p = 0.067 and p = 0.358, respectively). Other variables, including the age of the caregiver, educational status and relationship of the recipient, did not show statistically significant associations with the level of burden (p > 0.05).

**Table 4:** Association between Level of Perceived Burden and Gender, Employment and Type of Family.

Variables	Level of perceived burden			COR (95%CI)	AOR (95%CI)	p-value (AOR)
	Little or no to mild burden (n = 93)n(%)	Moderate to severe burden (n = 36)n(%)	Chi Square (p-value)			
***Caregivers***						
**Age**						
≤38	50(53.8)	14(38.9)	5.172 (0.160)			
>38	43(46.2)	22(61.1)				
**Gender**						
Male	57(61.3)	13(36.1)	6.630 (0.01*)	2.80(1.26-6.21)	0.44(0.18-1.05)	0.067
Female(Ref)	36(38.7)	23(63.9)		1	1	
**Educational Status**						
Up to Secondary	70(75.3)	32(88.9)	2.909 (0.088)			
Above Secondary	23(24.7)	4(11.1)				
**Employment**						
Employed	44(47.3)	10(27.8)	4.069 (0.044*)	2.33(1.01-5.38)	0.65(0.25-1.63)	0.358
Unemployed	49(52.7)	26(72.2)		1	1	
**Type of family**						
Nuclear	22(23.7)	18(50.0)	8.420 (0.004*)	0.31(0.13-0.69)	2.86(1.24-6.61)	0.013*
Joint	71(76.3)	18(50.0)		1	1	
**Relationship with care recipients**						
Spouse or children	63(67.7)	29(80.6)	2.083 (0.149)			
Others^#^	30(32.3)	7(19.4)				
***Care-recipient***						
**Age**						
60-75years	64(68.8)	30(83.3)	2.766 (0.096)			
≥75years	29(31.2)	6(16.7)				
**Duration of Mental Illness**						
≤2years	43(46.2)	15(41.7)	0.219 (0.540)			
>2years	50(53.8)	21(58.3)				

N = 129.

# Others (daughter-in-law, son-in-law, siblings, grandchildren); *P value significant at <0.05;

COR-Crude Odds Ratio; AOR-Adjusted Odds Ratio; Hosmer and Lemeshow Test:0.865;

Nagelkerke R Square: 0.191; Cox and Snell R Square:0.104.

Table 5 shows that there is no significant correlation between coping strategies and burden. However, the direction of relationship indicated that problem focused coping has inverse correlation with burden (r = −0.14; p = 0.112). In contrast, emotion focused coping showed a mild positive correlation with burden (r = 0.16; p = 0.057), and avoidant coping demonstrated a weak positive correlation with burden (r = 0.15; p = 0.074).

**Table 5:** Pearson Correlation between Problem Focused, Emotion Focused, Avoidant Coping and Burden.

Variables	Problem Focused Coping	Emotion Focused Coping	Avoidant Coping	Burden
**Problem Focused Coping**	1			
**Emotion Focused Coping**	0.675(0.000)**	1		
**Avoidant Coping**	0.221(0.012)*	0.291(0.001)**	1	
**Burden**	−0.141(0.112)	0.168(0.057)	0.158(0.074)	1

N = 129

**Correlation significant at P-value 0.01 level (2-tailed); *P-value 0.05 level (2-tailed).

## Discussion

This study aimed to assess coping strategies and perceived burden among caregivers of frail elderly with mental disorders.

In terms of coping strategies, caregivers in this study primarily utilized emotion focused coping, followed closely by problem- focused strategies. The preference for emotion focused coping (33.16 ± 4.917) suggests that caregivers may prioritize managing their emotional response to the stress of care-giving, perhaps due to the chronic and often irreversible nature of mental disorders in the frail elderly.

These results align with the findings of a cross-sectional study conducted in BPKIHS, Nepal which also noted a high reliance on both problem focused and emotion focused coping strategies [29].

Regarding perceived caregiver burden, nearly half of the respondents (48.1%) reported little or no burden, while 24.0% experienced mild to moderate burden, 12.4% experienced moderate to severe burden, and 15.5% reported severe burden. These findings contrast with research from rural China, where a significantly higher proportion of caregivers reported mild (40.2%) or moderate (39.5%), with only 7.4% reporting little or no burden [30]. Similarly, a descriptive co-relational study in Egypt involving 150 caregivers found that majority (44.6%) experienced a high level of burden [31], further highlighting the variations in caregiver experiences across different geographical and cultural contexts.

These contrasting findings may be explained by cultural and societal factors. In non-Western societies, caregivers-particularly adult children-often feel a strong sense of obligation to care for their ill parents, especially frail elderly. Additionally, strong societal and cultural norms that emphasize family responsibility may lead caregivers to perceive care-giving as a normal and important duty [32] which may reduce the likelihood of reporting high levels of burden.

Regarding factors associated with the level of perceived burden, gender was found to be a statistically significant factor in the level of perceived burden (𝑋^2^ = 6.630; p = 0.01). A higher proportion of female caregivers (63.9%) experienced moderate to severe burden compared to their male counterparts (36.1%). In the bivariate analysis, male caregivers were 2.8 times more likely to report lower levels of burden compared to females (COR  = 2.80; 95% CI: 1.26–6.21).The significant association between gender and burden observed here mirrors findings from studies in Nepal (𝑋^2^ = 5.9; p = 0.01) [33] and Egypt (𝑋^2^ = 16.3; p = 0.0001) [31]. However, our findings contrast with a study where gender was not a significant factor [34]. The correlation in the literatures is often justified by the traditional gender roles prevalent in the societies. Women often occupy the role of the primary caregiver, inheriting a “double burden” of managing intensive clinical care for a frail elderly while simultaneously fulfilling traditional domestic responsibilities. This multifaceted role can lead to physical and emotional exhaustion. However the discrepancy might suggest that as societal structures shift-particularly in urban settings-care giving responsibilities may be becoming more distributed, or that the specific mental health needs of the frail elderly in our sample placed a unique strain on one gender over the other. Similarly, employment status also showed a significant association with the level of burden during bivariate analysis (𝑋^2^ = 4.069; p = 0.044), which is consistent with the findings of the study conducted in India (p = 0.034f) [35]^33^ and Egypt (𝑋^2^  = 23; p = 0.001) [31]. A larger percentage of caregivers reporting moderate to severe burden were unemployed (72.2%) compared to those who were employed (27.8%). The crude odds ratio suggest that employed caregivers were 2.33 times more likely to report little or no burden compared to those who were unemployed (COR = 2.33; 95% CI: 1.01–5.38).

The high burden among unemployed caregivers can be justified by the severe financial pressure associated with caring for the frail elderly. While employment provides financial resources, it also results in “time –squeeze”-where the caregiver has fewer hours to dedicate to the patient’s complex needs, such as medication management and monitoring for safety. In contrast, some cross-sectional studies in Nepal reported no significant association between employment status of caregivers and caregiver burden [29,34]. This variation might be explained by differences in the socioeconomic status of the participants or the availability of secondary caregivers who can step in while the primary caregiver is at work. In the present study, type of family was found to be statistically significant correlated with level of burden (𝑋^2^ = 8.420; p = 0.004). Specifically, caregivers living in nuclear families were 2.8 times more likely to experience moderate to severe level of burden compared to those in joint families (AOR = 2.86; 95% CI = 1.24–6.61). This disparity can be attributed to the lack of a shared support system in nuclear households. In a joint family setting, the physical, emotional, and supervisory tasks required for a frail elderly person with mental disorders are often distributed among multiple family members. In contrast, those in nuclear families often bear the responsibility alone, leading to increased isolation and exhaustion. These results are supported by an observational analytical cross-sectional study in India, which found a higher prevalence of moderate to severe burden among nuclear family caregivers (77%) than those in joint families (68%) [36]. However, this finding contrasts with a descriptive cross-sectional study conducted in Nepal, which reported no significant association between the type of family and the level of caregiver burden [18].

There was no significant correlation between coping strategies and overall burden. A similar observation was reported in a descriptive cross- sectional study conducted among 102 caregivers of patients including frail elderly with mental disorders, at Chitwan Medical College, Nepal [18] The alignment of these results may be attributed to the similar demographic profiles of the care-givers in both studies, particularly the comparable median age range (38−40 years). In contrast, research from Nigeria reported a significant inverse relationship between caregiver burden and coping strategies (r = −0.58; p = 0.01) [37]. This difference may be attributed to varying cultural perceptions of care-giving, as caregivers in some societies hold strong traditional beliefs that caring for frail elderly family members is an inherent family duty, regardless of the specific coping strategy employed [38].

### Limitations

This study has certain limitations. Recruitment of participants from a hospital setting may introduce potential selection bias, as the sample may primarily represent individuals who seek hospital-based care and may not fully reflect the characteristics of the broader community population. There may also be the risk of response bias using interview technique such as social desirability bias by which caregivers either exaggerate or minimize their burden for some reason.

Furthermore, a predetermined structured interview could not investigate many elements related to coping and perceived burden among caregivers of frail elderly with mental disorders. Similarly, continuous variables were categorized into groups based on inter-quartile range to facilitate categorical analysis and clinical interpretation. While this approach allows for the identification of specific risk groups and ensures comparability with existing regional literature, it may result in a loss of data sensitivity compared to linear regression analysis of continuous scores.

Additionally,caregivers’ answers during data collection may have been impacted by the rush and crowd in psychiatric OPD. Similarly, duration of mental illness and duration of care-giving includes vast differences ranging from 6 months to 28 years. The variation in data might influence the level of perceived burden. Despite extending a week for data collection, the required sample size meeting inclusion criteria could not be met, which may also affect the result of the study.

### Recommendation

Considering the limitations of the study, study can be conducted in different settings (e.g., community settings), apart from hospital setting to minimize potential selection bias representing broader community participants/ caregivers with large sample size and probability sampling technique. Further, qualitative research can also be done to explore factors associated with caregiver burden experienced by caregivers of frail elderly with mental illness.

Similarly, self administered tool could be used to minimize socially desirable response bias.

Additionally, data collection could be done in the area comfortable to the respondents in order to gain more information regarding coping and perceived burden among them. Health professionals including nurses can help lessen caregiver burden by enhancing the ability to use of adaptable and effective coping strategies among caregivers especially those living in nuclear family, by assessing their needs, providing necessary psychological support, and intervening promptly.

### Implications

The findings of this study highlight the importance of strengthening family- centered mental health care in both nursing education and clinical practice. In nursing education, the results emphasize the need to incorporate comprehensive training on caregiver burden, psychosocial assessment, and family-centered mental health care into the nursing curriculum. Nursing students should be trained to assess the emotional, social, and psychological needs of caregivers and to develop effective communication and counseling skills to support families caring for individuals with mental illness. Integrating case-based learning and community exposure related to caregiver support can also help prepare future nurses to address these challenges effectively.

In terms of health practice, health professionals including nurses play a vital role in Identifying caregiver stress and providing appropriate guidance and support. Nurses working in psychiatric settings should routinely assess the well-being of caregivers, offer counseling and education about the illness, and connect caregivers with available support services. Additionally, health care institutions should consider developing caregiver support programs and psycho-educational interventions to reduce caregiver burden and improve the overall quality of care for both patients and their families.

## Conclusion

The study concludes that the utilization of both emotion- focused and problem- focused coping strategies plays a vital role in mitigating the perceived burden among caregivers of frail elderly with mental disorders. While nearly half of the participants reported minimal burden- potentially due to cultural norms of moral duties and responsibilities- those experiencing moderate to severe levels primarily faced significant personal strain. Furthermore, the intensity of this burden is significantly influenced by socio- demographic factors, particularly gender, employment status, and type of family.

The finding that caregivers in nuclear families face a higher risk of severe burden highlights a critical gap in social support, suggesting that community based interventions should prioritize caregivers lacking the protective buffer of an extended or joint family system.
